# The Mediating Effect of Social Problem-Solving Between Perfectionism and Subjective Well-Being

**DOI:** 10.3389/fpsyg.2021.764976

**Published:** 2021-12-10

**Authors:** Cheng Wang, Yisi Huang, Yueting Xiao

**Affiliations:** Department of Social and Behavioural Science, City University of Hong Kong, Kowloon, Hong Kong SAR, China

**Keywords:** perfectionism, subjective well-being (SWB), social problem-solving, mediating, adaptive, maladaptive, Rational Problem Solving (RPS)

## Abstract

This study examined the relationship between perfectionism and subjective well-being (SWB) and dimensions of social problem-solving ability. The Almost Perfect Scale-Revised (APS-R), Social problem-solving Inventory-Revised (SPSI-R) and Satisfaction with Life Scale (SWLS) were used to conduct a questionnaire survey of 202 Chinese adults. The results found that: (1) Subjective well-being was significantly negatively correlated with the discrepancy dimension of perfectionism and was also significantly negatively correlated with the negative problem orientation of social problem-solving. (2) The discrepancy dimension reflected in maladaptive perfectionism was significantly positively correlated with the negative aspects of social problem solving (Negative Problem Orientation, NPO; Impulsivity-Carelessness Style, ICS; and Avoidance Style, AS). (3) The negative problem orientation (NPO) dimension in SPSI-R, as a partial mediator, mediated the negative correlation between maladaptive perfectionism and subjective well-being. In conclusion, perfectionism and social problem-solving ability had different degrees of influence on the SWB of Chinese adults, and attention could be paid to dealing with discrepancy and how to reduce negative problem-solving tendency in education and clinical practice.

## 1. Introduction

Perfectionism seems to handicap the mental healthy, quality of life and happiness of individual (Hill et al., 2010). Many studies have shown that perfectionism is linked to mental illness, such as anxiety, depression, obsessive-compulsive disorder, and eating disorders (Frost and Steketee, 1997; Bardone-Cone et al., 2007; Williams and Levinson, 2020). There was also a high correlation with suicide (Hewitt et al., 1992). Thus, it is critical to investigate the underlying mediators between perfectionism and subjective well-being (SWB) to improve an individual's mental health.

Perfectionism could be defined as setting exceptionally high personal standards or expectations of actual performance with the judgement about one's self-worth based on the ability to meet these criteria (Burns, 1980; Fong and Yuen, 2014). Evidence have confirmed that perfectionism consists of both maladaptive and adaptive aspects (Parker and Adkins, 1995; Wang and Zhang, 2017). Perfectionism consists of three dimensions, Discrepancy, High Standards and Order (Slaney et al., 2001). The gap between unreachable standards and actual performance leads to accompanying stress and creates a discrepancy which represents maladaptive perfectionism (Suddarth and Slaney, 2001; Wang and Zhang, 2017). The other two factors, High Standards and Order, indicate adaptive perfectionism in that realistic goals match individual abilities bringing about the feelings of accomplishment and satisfaction (Suddarth and Slaney, 2001).

Maladaptive perfectionism hurts personal happiness, while adaptive perfectionism is conducive to personal development (Rice and Preusser, 2002; Gnilka et al., 2013). Strong associations between perfectionism and well-being were found in previous studies (Frost et al., 1993; Chan, 2007; Stoeber et al., 2020). Maladaptive perfectionism is negatively correlated with well-being (Suddarth and Slaney, 2001). In contrast, adaptive perfectionism tend to be positively correlated with higher levels of life satisfaction (Chang et al., 2004; Gilman et al., 2005) and self-esteem (Rice et al., 1998; Ozbilir et al., 2015).

Social problem-solving ability is inseparable from daily life, and research has shown that problem-solving ability has a strong correlation with the mental health of perfectionists (Chang, 2002). Social problem solving was defined as the process of solving problems encountered in social life. In the process of cognition, people found suitable methods to deal with daily problems (D'Zurilla and Nezu, 2010). Studies have shown that the way to deal with the problem has a certain impact on subjective well-being (Ben-Zur, 2009; Dursun, 2012). Good problem solving skills are efficacious in relieving mental tension in patients (Nezu and Perri, 1989; Sachs, 1991; Cheng, 2001) and play a positive role in subjective well-being (Suldo et al., 2011). Research on the relationship between Chinese college students' social problem-solving ability and SWB found that college students' social problem-solving ability was significantly correlated with SWB, and the combination of positive or negative problem-solving tendencies significantly predicts the SWB of college students (Liu, 2015). Another study on Chinese college students' coping styles and SWB showed that the more problem-solving and help-seeking behaviors college students display, the higher the level of positive emotions (Ling, et al.). On the contrary, negative coping styles are not conducive to individual problem solving and hurt people's physical and mental health (Sachs, 1991). Furthermore, a longitudinal study of Chinese adolescents showed that positive and rational problem-solving styles (PPO and RPS) were positively associated with SWB, while negative, impulsive and avoidant types of problem solving (NPO, ICS and AS) were negatively associated with SWB (Jiang, 2015).

Flett et al. (1996) found that maladaptive perfectionism was associated with more negatively oriented problem solving through cognitive, emotional and behavioral responses. The significant negative correlation between perfectionism and positive solutions to social problems was consistent with the findings of several previous studies on the relationship between perfectionism and severe psychological discomfort (Chang, 1998). Those with perfectionism and low problem-solving skills exhibit significantly more suicidal ideation compared to those with high problem-solving skills (Chang, 1998, 2002). In an evidence-based study of college students, it was noted that a perfectionists level of focus on social expectations was predictive of positive problem-solving orientations and the use of rational problem-solving methods. According to this study, the more that a perfectionist focuses on fulfilling social expectations, the lower their propensity to employ rational problem-solving strategies (Rich and Bonner, 2004).

Previous studies have shown that there are many intermediaries between perfectionism and SWB, such as self-compassion or compassion (Stoeber et al., 2020), self-presentation (Mackinnon and Sherry, 2012), self-identity (Yi, 2012) and self-esteem (Rice et al., 1998). Also, some studies have indicated that social problem-solving plays a mediating role between perfectionism and mental health (Besser et al., 2010). However, there were few empirical studies on whether social problem-solving plays an intermediary role between perfectionism and SWB. In the case of perfectionism, it is possible that the different ways in which people respond to problematic situations could lead to different levels of well-being. Therefore, this study proposes the hypothesis that social problem-solving ability plays a mediating role between perfectionism and SWB, that is, people with positive and rational social problem-solving attitudes have higher SWB, while people with negative, impulsive-negligent and avoidant social problem-solving attitudes have lower SWB. The purpose of this research is to study the mediating effect of social problem-solving between perfectionism and SWB. In summary, if social problem-solving had a mediating effect on the relationship between perfectionism and subjective well-being, improving problem-solving ability could be used as an intervention for the maladaptive form of perfectionism to reduce the chances of developing a mental disorder.

## 2. Methods

### 2.1. Participants and Procedure

Participants were 202 Chinese young people, including 142 women and 60 men, with an average age of 24.52 years (*SD* = 3.26) and a range of 18–38 years old. Participants were recruited through the social network WeChat, which was commonly used by Chinese people. Participants should have met the following inclusion criteria: (i) above 18 years old; (ii) completed all questionnaires within the specified time frame and had no missing data. Exclusion criteria: (i) minors; and (ii) participants with incomplete questionnaires. Before filling out the questionnaire, participants have been informed that the study involves a series of psychological variables related to perfectionism. It was recommended to complete this questionnaire in about 5–10 min. Participants participated in the questionnaire survey anonymously and voluntarily, and could withdraw from the survey at any time. In addition, all the data in the questionnaire were only be used for this research to ensure the privacy of the information. A total of 208 questionnaires were returned, and six questionnaires that did not answer the questions completely were excluded. The final number of valid questionnaires was 202.

### 2.2. Measures

#### 2.2.1. Almost Perfect Scale-Revised(APS-R)

The Chinese version of the Almost Perfect Scale(APS-R) used in this study is based on the original English version created by Slaney et al. (2001) and consists of 23 questions (Wang et al., 2007). The scale consists of three dimensions, High Standards(7 questions), Order (4 questions) and Discrepancy (12 questions), with Cronbach's α of 0.82, 0.68, and 0.88, respectively. The scale includes items such as “I have high standards for my performance at work or at school.” Participants are required to score according to the degree to which these descriptions are in line with themselves, using Likert 7-point scoring. The sum of the scores of all items in a dimension was the scores of participants in that dimension. APS-R usually divides people into adaptive and maladaptive perfectionists. Adaptable and maladaptive perfectionists get high scores in High Standards and Order, but maladaptive perfectionists also get high scores in Discrepancy. In this study, the internal consistency test was performed on all items, and the Cronbach's α of three dimensions, High Standards was 0.81, Order was 0.81, and Discrepancy was 0.92.

#### 2.2.2. Social Problem-Solving Inventory-Revised (SPSI-R)

This paper used a revised Chinese version of the questionnaire (SPSI-R) developed by Hong Kong scholars Siu and Shek (2005) on the basis of the English version developed by D'Zurilla et al. (2004) and finds it applicable to students in a Chinese cultural context. The scale is used to measure participants' ability to recognize particular problems encountered in everyday life and to find effective solutions. The English version of the scale has 52 items and contains five dimensions, namely Positive Problem Orientation (PPO), Negative Problem Orientation (NPO), Rational Problem Solving (RPS), Impulsive/Careless Style (ICS), and Avoidance Style (AS). The structure of the Chinese version of the questionnaire is essentially the same as that of the English version, covering a total of 32 items. Internal consistency coefficients for the five subscales reported in the text ranged from 0.64 to 0.89. Participants were asked to score these descriptions according to how well they fit or how much they agreed with the items, using a Likert 5-point scale. Higher total scores for subjects on both the PPO and RPS subscales indicated that they had better social problem solving skills and were better able to adapt to and face the challenges posed by their social environment. All items were tested for internal consistency and the Cronbach's α for the five dimensions, PPO was 0.72, NPO was 0.79, RPS was 0.87, ICS was 0.62, and AS was 0.89 in this study.

#### 2.2.3. Satisfaction With Life Scale (SWLS)

A scale developed by Diener et al. (1985) was used, which has 5 items. This scale was used to understand how satisfied the participants were with their lives and to reflect the individual's subjective well-being (SWB). The scale includes items such as “In most respects, my life is close to my ideal.” Participants were asked to score these items based on how well the descriptions fit them or how much they agreed with them, using a 7-point Likert scale. All items were scored using the positive scoring method. The higher the participant's total score on the 5 items, the greater their well-being and the more satisfied they were with their state of life. The Chinese version of the SWLS validated by Bai et al. (2011) was used in this study, which translated all the original items into Chinese and validated them through a standardized back translation procedure with a Cronbach's α of 0.88. The Cronbach's α for the total scale in this study was 0.87.

### 2.3. Statistical Analysis

Using SPSS21.0 and macro program PROCESS for data analysis, under the condition of controlling demographic variables such as age and gender, to perform reliability test, descriptive statistics, correlation analysis, regression analysis and mediation effect test on the data.

## 3. Results

### 3.1. Descriptive Statistics and Reliability of Each Variable

The descriptive statistics and internal consistency of each dimension of the SWB, APS-R and SPSI-R are presented in Table 1. The results show that all dimensions of APS-R and SPSI-R have good reliability, except for the dimension Impassivity/Carelessness Style (ICS) of the SPSI-R was 0.62, which was lower than 0.70.

**Table 1:** Means, standard deviations, Cronbach's a and range of scores for perfectionism, social problem solving, and subjective well-being (*N* = 202).

	**Mean**	**SD**	**α**	**Range**
APS-R Stnds	34.78	6.65	0.81	17–49
APS-R Ord	20.43	4.52	0.81	8–28
APS-R Disc	51.53	14.03	0.92	16–84
SPSI-R PPO	18.37	3.12	0.72	8–25
SPSI-R NPO	17.79	4.64	0.79	7–29
SPSI-R RPS	45.87	6.61	0.87	18–60
SPSI-R ICS	9.39	2.90	0.62	4–19
SPSI-R AS	12.09	4.70	0.89	5–24
SWB	20.76	6.34	0.87	5–35

*APS-R Stnds, Almost Perfect Scale-Revised–Standards subscale; APS-R Ord, Almost Perfect Scale- Revised-Order subscale; APS-R Disc, Almost Perfect Scale-Revised-Discrepancy subscale; SPSI-R, Social Problem Solving Inventory-Revised; SPSI-R PPO, Positive Problem Orientation Subscale; SPSI-R NPO, Negative Problem Orientation Subscale; SPSI-R RPS, Rational Problem Solving Subscale; SPSI-R ICS, Impulsivity/Carelessness Style Subscale; SPSI-R AS, Avoidance Style Subscale; SWB Subjective Well-being*.

### 3.2. Correlation Analysis of Various Variables

The results of the correlation between the variables are shown in Table 2. The Order dimension in APS-R significantly correlates with all five dimensions of SPSI-R; Positive Problem Orientation, PPO (*r* = 0.30, *p* < 0.01); Negative Problem Orientation, NPO (*r* = –0.17, *p* < 0.05); Rational Problem Sloving, RPS (*r* = 0.43, *p* < 0.01); Impulsivity/Carelessness style, ICS (*r* = –0.21, *p* < 0.01); Avoidance Style, AS (*r* = –0.30, *p* < 0.01). Specifically, the dimension Standards, which reflects adaptive perfectionism, is significantly positively correlated with the two positive dimensions of SPSI-R, Positive Problem Orientation (PPO) and Rational Problem Solving (RPS), respectively (*r* = 0.45, *p* < 0.01; *r* = 0.49, *p* < 0.01). As expected, Discrepancy, which represents negative perfectionism, was positively correlated with the negative dimensions of SPSI-R, Negative Problem Orientation, NPO) (*r* = 0.47, *p* < 0.01), Impulsivity/Carelessness Style, ICS (*r* = 0.15, *p* < 0.05) and Avoidance style, AS (*r* = 0.31, *p* < 0.01). For the SWB, only the dimension Discrepancy in APS-R has a significant negative correlation with it (*r* = 0.37, *p* < 0.01), and the other two dimensions, Standards and Order, do not show significant correlation with SWB. Specifically, the two negative dimensions (NPO, AS) and a positive dimension (PPO) of SPSI-R are negatively and positively correlated with SWB, respectively. Among them, NPO have the strongest correlation with SWB (*r* = –0.35, *p* < 0.01).

**Table 2:** Intercorrelations amongst measures of perfectionism, social problem solving and subjective well-being (*N* = 202).

	**1**	**2**	**3**	**4**	**5**	**6**	**7**	**8**	**9**
1. APS-R Stnds	-								
2. APS-R Ord	0.46^**^	-							
3. APS-R Disc	0.38^**^	0.15^*^	-						
4. SPSI-R PPO	0.45^**^	0.30^**^	0.02	-					
5. SPSI-R NPO	−0.04	−0.17^*^	0.47^**^	−0.38^**^	-				
6. SPSI-R RPS	0.49^**^	0.43^**^	0.00	0.53^**^	−0.26^**^	-			
7. SPSI-R ICS	−0.10	−0.21^**^	0.15^*^	−0.18^*^	0.25^**^	−0.39^**^	-		
8. SPSI-R AS	−0.12	−0.30^**^	0.31^**^	−0.19^**^	0.56^**^	−0.27^**^	0.45^**^	-	
9. SWB	0.06	0.09	−0.37^**^	0.30^**^	−0.35^**^	0.09	0.03	−0.16^*^	-

*APS-R Stnds, Almost Perfect Scale-Revised–Standards subscale; APS-R, Ord Almost Perfect Scale- Revised-Order subscale; APS-R Disc, Almost Perfect Scale-Revised-Discrepancy subscale; SPSI-R, Social Problem Solving Inventory-Revised; SPSI-R PPO, Positive Problem Orientation Subscale; SPSI-R NPO, Negative Problem Orientation Subscale; SPSI-R RPS, Rational Problem Solving Subscale; SPSI-R ICS, Impulsivity/Carelessness Style Subscale; SPSI-R AS, Avoidance Style Subscale; SWB, Subjective Well-being*.

* *p < 0.05 and*

** *p < 0.01*.

### 3.3. Mediation Analysis

**Figure 1:**
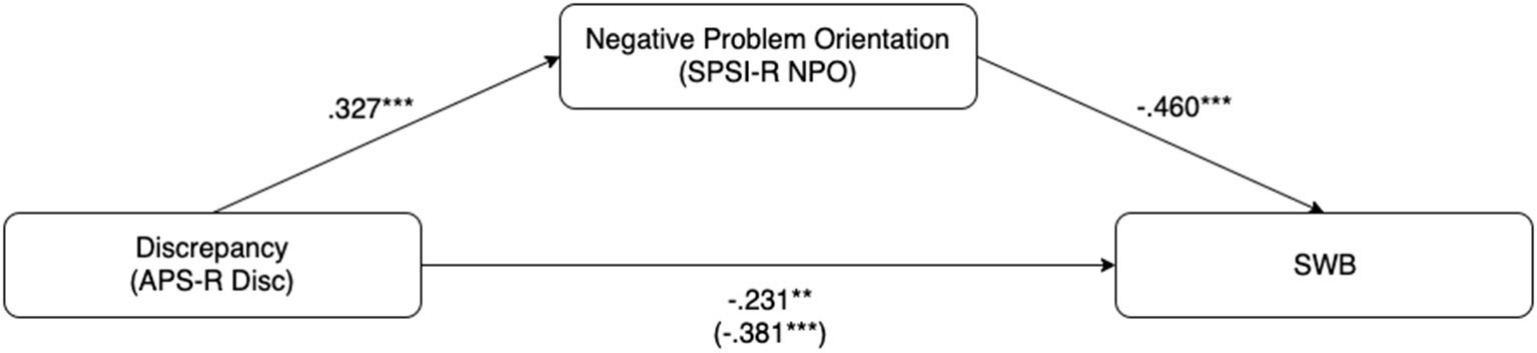
Mediation model of the relationship between APS-R Ord and SWB with SPSI-R RPS as a mediator. ^**^p < 0.01 and ^***^p < 0.001. APS-R Ord, Almost Perfect Scale-Revised-Order subscale; SPSI-R RPS, Social Problem Solving Inventory-Revised-Rational Problem Solving; SWB, Subjective Well-being.

The relationship between variables APS-Discrepancy, SPSI-NPO and SWB that are significant in both correlation and regression analysis are further explored, assuming SPIS-NPO as the mediator between APS-Discrepancy and SWB. Specifically, using the PROCESS macro for SPSS (Hayes, 2017), we tested a mediation model (model 4 in PROCESS) with APS-Discrepancy as the independent variable, SPSI-NPO as the mediator, and SWB as the dependent variable (see Table 3). Sex and age were entered as covariates. Providing support for the proposed mechanism of change, all paths of the mediation model were statistically significant (all *p* < 0.05; see Figure 1). The mediating effect test of SPSI-RPS was shown in Table 4. Indirect effect coefficient ab = –0.23 [95% confidence interval was (–0.24, –0.07)], c' = –0.23, *p* < 0.01 [95% confidence interval was (–0.39, –0.07)]. This result shows that SPSI-NPO has a partial mediation effect between APS-Discrepancy and SWB, that is, maladaptive perfectionism mainly affects the ability of negative problem solving, thereby affecting SWB.

**Table 3:** The mediation model among APS-R Disc, SPSI-R NPO and SWB (*N* = 202).

		***R***	***R^**2**^***	***F***	***b***	***SE***	***t***
SPSI-R NPO	APS-R Disc	0.51	0.27	23.95	0.33	0.04	8.00^***^
SWB	APS-R Disc	0.46	0.21	1.30	−0.23	0.08	−2.88^***^
	SPSI-R NPO				−0.46	0.12	−3.79^***^

*APS-R Ord, Almost Perfect Scale- Revised-Order subscale; SPSI-R RPS, Rational Problem Solving Subscale; SWB, Subjective Well-being*.

*** *p < 0.001*.

**Table 4:** Mediation Effect of APS-R Disc, SPSI-R NPO and SWB (*N* = 202).

	**Effect**	**BootSE**	**BootLLCI**	**BootULCI**	**Effect ratio (%)**
Total effect	–0.38	0.07	–0.52	–0.24	
Indirect effect	–0.15	0.04	–0.24	–0.07	39
Direct effect	–0.23	0.08	–0.39	–0.07	61

## 4. Discussion

The present study expanded upon previous research on perfectionism and subjective well-being (Chan, 2007; Mackinnon et al., 2017; Ko et al., 2020; Stoeber et al., 2020). As proposed in the hypothesis of the current study, the mediating role of problem-solving has been confirmed to some extent. The results suggested that the NPO dimension of social problem-solving mediated between the Discrepancy dimension of perfectionism and SWB. Contrary to expectations, no mediating role for social problem-solving was found for any of the other dimensions of perfectionism and SWB. Additionally, this survey investigated the links between perfectionism, social responsibility and solving social problems. Unsurprisingly, maladaptive perfectionism (Discrepancy) was significantly and negatively associated with SWB, which was in agreement with previous studies, while unlike previous research experiences, adaptive perfectionism (High standards and Order) had a positive but non-significant relationship with SWB (Bulina, 2014; Suh et al., 2017; Karababa, 2018; Fallahchai et al., 2019). Also, research has shown that the NPO and AS dimensions of social problem-solving variable had a significant negative relationship with SWB, and the PPO dimension of social problem-solving had a significant positive relationship with SWB (Elliott et al., 2001; Chang et al., 2009), which was confirmed by our study results. Unlike previous studies, which have shown that the style of acting and attitude of individuals in coping with stressful situations predicted SWB, the present study did not reveal significant relationships between the other three dimensions of problem-solving and SWB (Cosway et al., 2000; Suldo et al., 2011; Chen, 2016).

Furthermore, in conjunction with the mediation model, it could be concluded that perfectionists conforming to the discrepancy dimension would have a significant effect on their own SWB by influencing the social problem-solving of the Negative Problem Orientation dimension. Maladaptive perfectionists were more likely to choose negative ways of dealing with problems when faced with stressful events, such as avoiding the problem of coping with negative emotions and attitudes. They would be more inclined to strive for flawlessness, which is not feasible in real life, so they often censor themselves for facing “failure,” for example, “when faced with a difficult problem, I do not think I can solve it no matter how hard I try” (D'Zurilla et al., 2004). Furthermore, maladaptive perfectionists were more likely to feel ashamed of their failures and thus more likely to engage in maladaptive behaviors such as bad spending or even suicide (Brennan-Wydra et al., 2021; Chang et al., 2021). According to this logic, maladaptive perfectionist would be more likely to avoid problems in order to avoid failure, or often cope with stress in a negative way, which could lead to stress and anxiety if the task fails (Santanello and Gardner, 2007; Mofield et al., 2016). In contrast, adaptive perfectionists cope with problems without intense frustration, even if the problem has not been solved, and thus maintain a positive and healthy mental mood (Mofield et al., 2016).

As previously mentioned, not all outcomes were in alignment with previous studies, and in response to this, the following possible reasons and options for improvement could be put forth. First, appropriate extrapolation of the predictive role using relevant data needs to be further substantiated by longitudinal studies. Although it seems reasonable based on the hierarchical regression model and mediating model that the predictor was a personal trait in perfectionism, the mediator is the attitude of problem-solving, and the dependent variable is the state of individual subjective well-being, which may lead to a predictive path in the opposite direction (e.g., the individual's current SWB influences the trait of perfectionism through their attitude toward problem-solving). Researchers have shown that human characteristics are constantly changing in adulthood due to environmental changes or life incidents (Hoffman, 1991; Bleidorn et al., 2018; Woods et al., 2019). Second, the unbalanced gender ratio of subjects in this study may have contributed to inconsistency in the findings with past studies in some respects (e.g., no correlation was shown between adaptive perfectionism and SWB). Previous studies have confirmed that women have a higher level of maladaptive perfectionism than men (Haase et al., 2013), and the 70% female subjects in this study could lead to bias in the findings. Therefore, in subsequent studies, balancing the proportion of male and female subjects could increase credibility of the results. Last, as the questionnaire has 62 questions and took a long time for subjects to fill in, it may lead to a loss of patience in the process in filling in the questions and not reading them carefully before answering, thus making the data portray an inaccurate picture (Farooq, 2018; Krosnick, 2018). Therefore, the selected questionnaire could be appropriately screened to makes it shorter and tested for validity and reliability before administering.

So far, SWB has received extensive attention from scholars (Diener and Suh, 2000; Diener, 2009; Blanchflower, 2021; Möhring et al., 2021). Studies have shown that during the COVID-19 pandemic, university students' well-being was affected, and they felt more symptoms of stress and mood disorders than before (Charles et al., 2021). A study in China showed that students affected by COVID-19 felt more psychological stress due to the impact of prolonged suspensions (Wang et al., 2020). Besides, research has shown that adaptive perfectionists set themselves realistic goals before a task, use their skills and potential to the fullest, look inwards when they don't achieve their goals, and feel less stress as a result (Vansteenkiste et al., 2010; Eum and Rice, 2011). A study of Chinese college students studying in the United States showed that maladaptive perfectionism predicted more symptoms of depression and anxiety (Liu et al., 2021). There is much evidence that maladaptive perfectionism not only occurs in mental disorders (Pinto et al., 2017; Hu et al., 2019; Levine et al., 2019), but is also a risk and maintenance factor for a range of problems (Lo and Abbott, 2013). Therefore, interventions that target maladaptive perfectionism in the treatment of these disorders may lead to better outcomes.

In summary, although there is much room for further exploration and refinement in this study, it is worth acknowledging that, based on the results of this experiment and past research experience, the following recommendations could be made for personal development and mental health. On the one hand, schools, units and communities could offer relevant courses and training to reduce the negative problem solving of people in times of stress, in other words, to develop the ability to think rationally and solve problems. For example, schools could develop students' problem-solving skills through STEM courses, etc., thus contributing to the future development of the individual (Simamora et al., 2017; Mutakinati et al., 2018; Özreçberoğlu and Çağanağa, 2018; Fülöp, 2021). On the other hand, since the adaptive perfectionist sets high standards for themselves, and since the maladaptive perfectionist often sets the standards too high and fails, so having the right self-concept could lead to a reduction in stress and a sense of well-being (Zimmer-Gembeck et al., 2018; Miller et al., 2019).

## Data Availability Statement

The raw data supporting the conclusions of this article will be made available by the authors, without undue reservation.

## Ethics Statement

The studies involving human participants were reviewed and approved by Ethic Review Board of City University of Hong Kong. The patients/participants provided their written informed consent to participate in this study.

## Author Contributions

CW is the experimental designer and executor of this study. CW and YH have completed data analysis and written the first draft of the paper. YH participated in experimental design and analysis of experimental results. CW is the designer and person in charge of the project, guiding the writing and modification of the experimental design data analysis paper. CW and YX have participated in the discussion of reviewer's comments and also gave some useful suggestions for revision, and has participated in the second round of data collection and data analysis of the results. All authors contributed to revision, read, and approved the submitted version.

## Conflict of Interest

The authors declare that the research was conducted in the absence of any commercial or financial relationships that could be construed as a potential conflict of interest.

## Publisher's Note

All claims expressed in this article are solely those of the authors and do not necessarily represent those of their affiliated organizations, or those of the publisher, the editors and the reviewers. Any product that may be evaluated in this article, or claim that may be made by its manufacturer, is not guaranteed or endorsed by the publisher.
